# Barriers and facilitators to achieving optimal oral health behaviours in university students

**DOI:** 10.1186/s12889-025-25042-8

**Published:** 2025-11-10

**Authors:** Tanzeelah Azam, Michaela Goodwin, Juliana Gomez, George Kitsaras

**Affiliations:** 1 Dental Health Unit, University of Manchester, Lloyd Street North, Manchester, M15 6SE UK; 2 Dental Health Unit, Colgate-Palmolive, Lloyd Street North, Manchester, M15 6SE UK

**Keywords:** Oral health, Barriers, Facilitators, Behaviours, University students, Theoretical domains framework, Behaviour change techniques, Behaviour change wheel

## Abstract

**Background:**

There is little research regarding how oral health behaviours in young adults change during their time at university. Engaging in behaviours that increase the risk of oral health diseases may have life-long consequences in this demographic, including their oral health and overall well-being. This study aimed to understand the oral health behaviours of university students - including toothbrushing, flossing, sugar consumption and dental visits - and explore the barriers and facilitators that influenced the establishment and maintenance of optimal oral health behaviours.

**Methods:**

Participants were recruited across the University of Manchester through emails, university websites, and posters. Subsequently, qualitative semi-structured interviews based on the Theoretical Domains Framework (TDF) were conducted with 21 students comprising a mix of males and females aged between 18 and 24 years old, in their first year of university.

**Results:**

Employing a deductive approach, the participant’s statements were systematically categorised into the 14 domains within the TDF. Five intervention functions outlined in the Behaviour Change Wheel (BCW), and 23 Behavioural Change Techniques (BCTs) were deemed pertinent and recognised within the context of this study. Primary obstacles that hindered the adoption of ideal oral health behaviours encompassed limited knowledge, physical and cognitive fatigue, busy schedules during exam periods, financial constraints impacting the purchase of dental products and healthy dietary choices and the cost of private dental treatment. Conversely, factors that facilitated the adoption of optimal oral health behaviours included positive intentions, strong and steadfast goals, and beliefs surrounding consequences.

**Conclusion:**

The outcome of this qualitative study provided valuable information regarding the barriers and facilitators to achieving optimal oral health behaviours in young adults at university. This facilitated the identification and recognition of areas necessitating further support for this population and pinpointed the required areas for change. By utilising the TDF and BCW, tailored interventions can be designed to address oral health behaviours. The gathered data directly informs the development of future behaviour change interventions, targeting improvements in oral health and general health behaviours in this population.

## Background

Throughout their university experience, students often transition from late adolescence to young adulthood, a period often characterised by exploration and numerous changes [1, 2]. Students undergo a process of increased independence, with some gaining independence for the first time in their lives [3, 4]. This newfound self-reliance, coupled with the formation of new peer networks and a fresh perspective on life, may contribute to the adoption of new behaviours, potentially including risk behaviours, such as smoking and substance use [5]. Consequently, university students may initiate lifelong behaviours, alter current behaviours and undertake risks [6]. Many risk-related behaviours are related to oral disease and can have a damaging consequence on the oral health of an individual. Behaviours can include smoking, stress, poor oral hygiene [7], poor dietary habits [8], infrequent dental visits [9], drug or substance abuse [10, 11], and alcohol consumption [12]. Engaging in these behaviours can impact individuals, potentially resulting in future long-term illnesses [13]. Given that university students are prone to engaging in unhealthy behaviours [14, 15], it is important to acknowledge this, as implementing behaviour change becomes increasingly challenging later in life [16]. Given the exposure to risk-related behaviours among university students, it is important to understand the barriers to adopting healthy habits including good oral hygiene routines. Currently, there is little research using theory-based approaches for identifying the barriers and facilitators present for university students in changing oral health behaviours at university. Oral health behaviours encompass oral hygiene routines, dental service use and nutritional preferences which can impact individual oral health [17]. Addressing this area will aid in mitigating the impact of risk behaviours on oral health in this under-researched population.

The health of the mouth is crucial for the overall health of the body [18]. It is suggested that young adults can be at risk of oral diseases [19]. This is because, in this demographic, between 44% and 57% of university students may have dental decay [20, 21] and 32.8% of university students do not brush their teeth twice a day [22]. Furthermore, university students can adopt unhealthy eating habits [1] that can affect oral health [23]. In addition, only 4.7% of young adults have a healthy periodontium [24] and only 14.2% of individuals aged 21 years or less have been shown to use preventative dental programmes or services [19]. Therefore, it is important to ensure that the oral health of students is understood and addressed during their time at university.

This study addresses evidence gaps by utilising the Theoretical Domains Framework (TDF) [25] in conjunction with the Behaviour Change Wheel (BCW) [26]. The TDF, established by Michie and colleagues, is grounded in behavioural psychology [27] and consists of 14 domains that help understand the determinants of behaviour and barriers to change [28, 29]. It underlies the COM-B model (Capability, Opportunity, and Motivation for Behaviour model), offering a fine-grained approach to exploring these determinants. Positioned within the BCW, the TDF helps identify appropriate intervention functions and the corresponding Behaviour Change Techniques (BCTs) [25]. These BCTs serve as components of an intervention designed to change behaviours [28]. An intervention can then be formulated to tackle the barriers while supporting facilitators [28]. Using the TDF allowed the identification of barriers and facilitators [25] to achieving optimal oral health behaviours in first-year university students, laying the groundwork for targeted interventions.

The BCW serves as a comprehensive guide to strategies for modifying behaviour, helping to recognise what needs to change for the target behaviour to occur [26] and linking the determinants identified by the TDF to specific BCTs [30]. Developed as a synthesis of 19 behaviour change frameworks [31], and 33 behaviour change theories [32], the BCW centres around components including intervention functions and the COM-B model. There are nine intervention functions that incorporate BCTs to drive behaviour change [31]. The BCW focuses on the COM-B model to acknowledge the necessary elements of the target behaviour, to help identify what needs to change, for whom, when, where and how to support students in improving their oral health behaviours, focusing on the identified barriers and facilitators [31]. Based on the COM-B model, changes in behaviours occur when an individual has the capability, opportunity, and motivation to engage with the target behaviour and sustain changes over time. Capability is broken down into physical and psychological [1], opportunity into physical and social, and motivation into reflective and automatic motivation [33]. These three elements of the COM-B model are intertwined.

The TDF and BCW offer a theoretical and structured foundation for understanding and changing behaviour [26, 32]. These frameworks can be used to explore behaviours and develop interventions to promote healthy lifestyle choices [34].

### Aims and objectives

This study aimed to understand oral health behaviours and explore how to support university students to establish and follow optimal oral health behaviours. This aim was achieved through the following objectives: (a) exploring barriers and facilitators by utilising the TDF in a qualitative research project with university students, (b) using the BCW to explore what needs to change, when, where and how to support students in improving their oral health behaviours, focusing on the identified barriers and facilitators (c) identifying specific BCTs that need to be incorporated in a future intervention to address the identified barriers and enhance the facilitators.

## Methodology

This was a qualitative semi-structured interview study involving university students based on the TDF and analysed using framework analysis.

### Recruitment

Sampling followed a convenience sampling approach. Semi-structured interviews were carried out with 21 participants (aged between 18 and 24 years old) studying in their first year at university. These participants volunteered and were recruited for the study based on their interest in participating. Selection of participants was made according to the following eligibility inclusion criteria: (a) the ability to comprehend and speak English, (b) first-year student at the University, (c) availability for the study duration, (d) ability to attend an interview on campus or online and (e) completion of an informed consent form. Participants were compensated for their time in the form of £10 vouchers for online shopping. The protocol and study design were reviewed and approved before the commencement of this study by the University of Manchester Research Ethics Committee (UREC) (Reference: 2023–15650-30363). Written consent was obtained by online consent forms sent via email during the recruitment process and all participants consented to the anonymous use of their data for analysis and subsequent publication. Data was collected on the participants’ degree subjects, with no exclusions based on their field of study. The majority (*n* = 20) were enrolled in non-medical/non-health degrees; therefore, this did not pose an issue regarding responses being influenced by the subject studied. Also, all students were in their first year and had not considerably advanced in their academic programs, which reduced the likelihood of any preconceived notions affecting their responses. The COREQ checklist was utilised to guide the reporting of this study, ensuring all critical aspects of the research process, including participant selection, data collection, and analysis were transparently reported [35] [see Additional file 5].

### Data collection

The interviews were directed by an interview guide [see Additional file 1] and were held over Zoom (video or call). The interviews were all completed by the same interviewer and there were no dropouts from this study. Patient and Public Involvement (PPI) work was completed in January-February 2022 to understand appropriate questions to be asked to develop an interview guide to address specific challenges and factors relevant to this population and which was grounded on the 14 TDF domains.

### Data analysis

Each audio-recorded interview was transcribed verbatim using a transcription service and analysed using the TDF framework. Each participant’s response was mapped with the TDF domains using a deductive approach by two independent coders. Barriers and facilitators were identified, and discussions were held if coders had disagreements. A third independent coder was available to address and resolve any further disputes; however, there was no need to utilise the third coder as there were no disagreements. Overarching themes were summarised, and frequency counts of the TDF domains were used to identify the main constructs and the most commonly supported TDF domains.

## Results

### Sample characteristics

A total of 21 participants, comprising 12 males and 9 females, engaged in this qualitative study exploring barriers and facilitators, with no dropouts. Their average age was 21 years old (SD = 1.54). The study’s participants were ethnically diverse, including White (*n* = 5), Asian or British Asian (*n* = 6), and Black, Black British, Caribbean, or African (*n* = 10).

### Inter-rater reliability

Cohen’s Kappa was calculated to assess the inter-rater reliability between the two independent coders. A total of 330 statements were examined and mapped into the TDF domains. The analysis revealed substantial agreement between the coders (k = 0.982), highlighting the effective application of reflexivity control, achieved through the involvement of an experienced interviewer and two independent coders.

### Overview of data saturation

Data saturation was monitored, and data collection ceased after the 21 st interview, as no new themes emerged. Most TDF domains were covered by participant’s responses, with the exception of the ‘social or professional role’ domain. Responses were mapped to at least 13 of the 14 domains.

### Barriers and facilitators by TDF domain

Below is a presentation of example quotations, focused on the examination of individual TDF domains, illustrating participant responses.

#### Knowledge

Some students sought more detailed brushing and flossing knowledge to enhance oral hygiene. They recognised the importance of oral hygiene, and the impact sugary foods and alcohol can have on teeth, often relying on social media and dental professionals for information.

*“… but I would really appreciate if I would gain more skills or knowledge on oral hygiene.” (P325)*.

*“… avoid sugary things and also the food stuff that they eat*,* they ensure they are helping their teeth to have a better condition… those people who maybe eat sugary things*,* take alcohol*,* I think their oral health is a bit lower. "* (P328).

#### Skills

Students highlighted the importance of accurate brushing techniques along with using the right products and demonstrating manual dexterity. While many maintained good oral hygiene practices, they recognised the need for better consistency in flossing. Challenges included mastering skills to access all areas of the mouth, with concerns about reach, coverage, and accuracy.

*“You need good dexterity and information on how to brush your teeth properly.”* (P003).

*“I think flossing is one of the skills I would wish to be able to improve…”* (P078).

*“…it’s just consistency at the moment… maybe incorporating more things by using a mouthwash*,* flossing will also help.”* (P099).

#### Belief about capabilities

Students shifted their perspective to take sole responsibility for their oral health but found it challenging to maintain routines during late nights or busy periods, particularly night-time brushing. While some remained consistent, others struggled due to busy schedules.

*“Continue taking care of them and be purposeful to be happy*,* not to remind on other people to remind me but do it because it makes me happy and boosts my self-esteem.”* (P329).

#### Optimism

Students were generally optimistic about their oral health and expressed confidence in the potential benefits of an oral health monitoring app. However, optimism varied, with past experiences and family history influencing their confidence levels.

“I am quite confident that they are healthy but it’s a journey, so I need to keep it that way. Because I have never been troubled with my teeth and they’re white, they’re strong, I chew things without feeling any pain… Yeah, I am quite good.” (P323).

“Potentially if app was well-developed, then I would be more likely to pick it up and use it.” (P254).

#### Belief about consequences

Students recognised that poor choices, such as consuming sugary or junk food, often led to adverse consequences, which were frequently followed by regret.

*“…students eat a lot more junk food*,* which means that they’re more likely to get cavities.” (*P003).

*“To prevent disease*,* this is my final goal…because it’s painful and it costs money.”* (P031).

#### Reinforcement

Since starting university, oral hygiene has become more important for many students, influenced by peer interactions, experiences of discomfort and the need for treatment. Neglecting toothbrushing led to discomfort, reduced confidence, and reluctance to engage socially. Despite this, students remained optimistic about preventing such lapses. Completing their routines provided a sense of achievement, whereas failure to do so resulted in disappointment.

“The oral hygiene has… It has changed in terms of it has increased. Because of interactions with other students. Also having good teeth makes you more attractive. Very important.” (P338).

*“It would be disgusting because I can’t even smile in public.”* (P326).

#### Intention

Students intended to improve their oral hygiene routines by making changes and adding new elements to achieve long-term benefits, such as disease prevention and reduced treatment costs. Each student demonstrated strong motivation to adapt their practices and showed optimism about their future oral health behaviours.

*“Hopefully I’ll be able to be more regular with my tooth brushing and…incorporate flossing more often.”* (P003).

*“It will be much better … after I’ve acquired good guidelines and what I’m required.”* (P328).

#### Goals

Students aimed to maintain a healthy mouth to prevent disease, cavities and pain in the long-term. They were also conscious of the impression their oral health made on others, striving to keep their mouths disease-free and avoid unnecessary pain and treatment.

*“I guess all the above whiter teeth*,* protect against disease…I continue to have healthy gums and good teeth for the sake of meeting people and making a good first impression…”* (P169).

#### Memory, attention, decision processes

The university workload affected students’ tiredness, impacting their oral hygiene. They sometimes forgot or neglected routines, especially at night, due to cognitive and physical exhaustion. Students prioritised quick brushing for cleanliness and omitted other steps, affecting brushing frequency, particularly during exams.

*“If you’ve been given a lot more work to do*,* I feel really tired by the end of the day*,* and then I sometimes just go to bed directly without brushing my teeth.”* (P003).

Students classified their brushing as either automatic or conscious efforts. For some, it was ingrained in their activities, while others viewed it as a conscious decision. Some fell in between, seeing brushing as a conscious decision that occasionally required reminders to ensure consistency.

*“I do have to remind myself*,* even though it’s an automatic feeling*,* usually it’s automatic…I need to remind myself*,* I need to put an alarm or something like that.”* (P329).

#### Environmental context and resources

Many students continued to visit their previous dentists due to concerns about registration, availability, and new practices not accepting new NHS patients. Cost also posed as a barrier, especially for private treatment, further impeding access to care.

*“I’ve not registered with a dentist*,* mainly because at the moment I feel like it’s very hard to register with a dentist. Most of them are not allowing new registration.”* (P003).

*“…I can’t afford right now. I mean like check-ups.”* (P324).

Some students preferred en-suite facilities or personal sinks in their rooms, finding shared bathrooms inconvenient and a potential barrier to maintaining their oral hygiene routines.

*“…when I had a room with a basin in it and that made brushing my teeth a lot easier than at the moment where I have a shared bathroom… it’s not too much of a distance but that little distance…it’s added an extra barrier.” (P099)*.

*“I’ve got an en-suite*,* so it makes it easier.”* (P013).

Students reported changes to their dietary habits since starting university, with some experiencing increased tooth sensitivity, possibly linked to these new dietary habits.

*“…When I wake up after drinking alcohol*,* my teeth feel really sensitive.”* (P145).

Most students felt capable of maintaining their oral hygiene, but some encountered challenges in accessing certain products such as floss or mouthwash. Financial constraints at university limited their ability to purchase certain dental products and healthier foods.

*“I would say floss is a little bit difficult to get hold of but toothbrush…”* (P325).

*“I think most they have the same routine because we lack the funds for a better oral health routine.”* (P324).

*“I would have a lot more extra materials*,* so like flossing*,* my mouthwash and just maybe being able to buy a more expensive food base which is healthier for my teeth.” (P078)*.

#### Social influences

Students commonly perceived their parents and friends as concerned about their oral health, viewing it as a reflection of self-care and independence. They valued the importance of others’ perceptions, particularly regarding tooth colour and appearance, which could influence their interactions. Consequently, they prioritised oral hygiene in social interactions to appear clean and hygienic.

*“Personally*,* my parents would never let me get cavities or gingivitis or any of the diseases from the mouth…I usually have conversations with a couple of friends… So*,* if I have a friend who isn’t brushing their teeth or isn’t flossing it’s my responsibility to tell them.” (P325)*.

*“Yeah*,* it’s very important. It’s very important…it really matters what others think about my oral health.”* (P328).

Many students noticed similarities among their peers in oral hygiene routines and the reasons behind occasional lapses, such as not brushing twice a day or skipping flossing. However, they recognised individual differences in routines and the varied choices of dental products used by different people.

*“I think definitely similar to other students’ routine because…I don’t think everyone has the time to do*,* you know*,* the full brushing and flossing.”* (P254).

#### Emotion

A student stated they felt depressed sometimes while at university so a system in place that would require them to brush their teeth or take care of themselves would help. This student was signposted and advised about the support available at the University.

*“There are times that I don’t really feel like brushing my teeth*,* I don’t really feel like flossing*,* I don’t really feel like doing anything*,* to be honest. Sometimes I wake up depressed*,* so I would say it’s really something that would help me out during the times when I’m down.” (P325).*

#### Behavioural regulation

Technology could help students adhere to oral hygiene routines, for example, through notifications and calendar reminders to bring a toothbrush when going out. Students recognised the need to brush their teeth after consuming certain foods, especially junk foods, due to their potential impact on teeth.

*“I think it’s easy. Yeah I think I have a notification to remind me come*,* it’s kind of easy with the technology…In case I am going out I set… I need to carry this and this and I have a list of things I carry when I go out.”* (P322).

*“I usually eat sugary foods and I love soda and all that*,* so I love junk food*,* so I feel like it has affected me so much since once I know I’ve eaten junk food today*,* I need to brush my teeth before I sleep.”* (P329).

### Barriers and facilitators

The barriers and facilitators identified from the domains and participants’ responses, regarding achieving optimal oral health behaviours during a student’s time at university, are shown in Table 1. An additional file shows this analysis in more detail [see Table 2 Additional file 2].

**Table 1:** Summary of the key barriers and facilitators to achieving optimal oral health behaviours in university students

Barriers:	Facilitators:
Lack of in-depth knowledge and skills – Some students had only basic brushing knowledge and were unaware of optimal timing, techniques, and methodology. Challenges in reaching all areas of the mouth were also reported.	Basic oral health knowledge – While some students lacked in-depth understanding, their foundational knowledge helped sustain basic oral hygiene habits.
University workload and lifestyle – Busy schedules, exam stress, and social activities disrupted oral health routines. Fatigue, forgetfulness, and lack of reminders also contributed to inconsistent oral hygiene practices.	Social influences and peer awareness – Observing peers’ oral health habits increased students’ awareness and motivation to maintain their own routines.
Financial constraints – Budget limitations influenced students’ ability to purchase dental products, opt for healthier diets, and access dental services.	Psychological and emotional benefits – Maintaining good oral hygiene contributed to a sense of accomplishment, self-confidence, and happiness.
Housing and bathroom arrangements – Shared bathrooms and lack of personal sinks created challenges in maintaining consistent oral hygiene routines.	Parental and peer reinforcement – Encouragement from family and friends played a role in reinforcing positive oral health behaviours.
Limited use of technology – Despite interest in oral health monitoring apps, students were not actively using such technology to support their habits.	Technological awareness – Students expressed optimism toward the potential of oral health monitoring apps or digital tools, viewing them as valuable resources for learning and habit formation.
Awareness of consequences but difficulty in implementation – Students were aware of the negative outcomes of poor oral health but struggled to consistently apply good habits due to competing priorities.	Future aspirations and awareness of consequences – Many students recognised the importance of oral health for social perception, professionalism, and long-term well-being, which motivated them to maintain good habits.

### Mapping and specifying the intervention functions

Five out of the nine intervention functions outlined in the BCW guide were deemed pertinent (education, training, enablement, persuasion, environmental restructuring) [see Table 2 in Additional file 3 for mapping of TDF domains, COM-B, and intervention function integration].

### Specifying the BCTs behaviour change techniques (BCTs)

The most suitable BCTs required to achieve optimal oral health behaviours in university students were identified through the insights gathered from the qualitative interviews to form the basis of a forthcoming intervention. A total of 23 BCTs were recognised from the interview data [see Table 3 in Additional file 4 for a more in-depth intricate relationships between the COM-B model, and TDF domains, including direct quotes from participants, intervention functions and BCTs]. These interconnections, derived from the study’s findings, revealed the complex associations in the context of behaviour change. This study did not reflect on the policy categories in detail as this was not deemed relevant for this study. The suitability of the delivery method for an intervention relies on the environment, the intended audience, and the specific behaviour. Further information regarding the taxonomy of the delivery methods is available in the BCW guide.

## Discussion

### Summary of key findings

This is the first study to investigate the barriers and facilitators to achieving optimal oral health behaviours among university students, with the aim of identifying specific intervention techniques to support this population in maintaining good oral hygiene, regular dental attendance, making informed nutritional choices and encouraging healthier oral care practices. Utilising the TDF and BCW in a qualitative study involving university students, we were able to gain a deep understanding of the factors influencing their oral health practices. Therefore, with this new understanding, targeted interventions and programmes can be developed and implemented, aimed at addressing these barriers and enhancing the facilitators. Such initiatives may have the potential to improve oral health outcomes for university students, ultimately leading to better long-term oral health.

The study identified key barriers, including a lack of in-depth knowledge and skills, university workload, exams, tiredness, lack of routines, insufficient reminders, social life, budget constraints, and housing arrangements (such as shared bathrooms). Conversely, the main facilitators included basic oral health knowledge, reinforcement from parents and peers, having a sink in their rooms, strong goals, positive intentions and commitment to future oral hygiene practices, and awareness of negative consequences. University students acknowledged their lack of comprehensive knowledge and skills for improving oral health behaviours but expressed readiness and motivation for behaviour change if necessary. Physical and cognitive fatigue, along with forgetfulness, hindered the consistent practice of oral hygiene routines and acted as barriers to achieving optimal oral health. Despite understanding the importance of their routines and the anticipated regret if missed, students often attributed tiredness, fatigue, and forgetfulness as reasons for missing their oral hygiene routines. This may have largely stemmed from their academic responsibilities [36], including exams and workload [37].

Students highlighted concerns regarding limited access to dental services, identifying it as a barrier. A study by Evans et al. (2023) supported this notion, indicating a decrease in accessibility to NHS dental services for patients and a growing number of dentists transitioning from the NHS to the private dental sector. Students identified several other barriers affecting their achievement of optimal oral health behaviours while at university, such as financial constraints impacting the purchase of dental products, likely due to other financial demands and priorities. As well as a lack of comprehensive information about oral health hindered improvements to their routines [38].

Some students reported that budget constraints and financial limitations impeded their ability to purchase and choose healthier food and drinks that may be less harmful to their teeth, be less detrimental to their overall oral health, and could have contributed to better oral health outcomes. Previous research highlighted the broader impact of financial constraints on fulfilling nutritional requirements. In the context of oral health, this implies that the financial burden may extend beyond affecting general nutrition to contributing to the impact on oral health [39]. The interaction between financial challenges and oral health underscores the need for targeted interventions. The cost associated with campus meal options may pose an additional financial burden on students already handling budget constraints. Also, cultural preferences and dietary choices such as among international students, minority groups or different ethnicities may not always align with the meal options available on campus, potentially leading to suboptimal dietary habits [40–42] and, consequently, may impact oral health [43]. Therefore, recognising and addressing challenges related to nutritional intake is essential for developing interventions aimed at promoting oral health among students.

The study findings suggested that students predominantly relied on social media platforms and information shared by dental professionals as their primary course of oral health information. This reliance on technology aligns with existing literature emphasising the technology and digital inclination in this population [44]. The trend among young adults involved utilising technology, including social media and apps, as a means to gather health-related information [45]. Young adults acknowledged the potential of digital platforms for accessing healthcare advice online, enabling them to understand their health conditions and bodies better. However, it was also noted that they valued direct face-to-face interactions with healthcare professionals for obtaining health information [44].

The study findings indicated that, in general, there was limited integration of technology into students’ oral health practices. This suggested that there might have been insufficient attention given to the use of digital tools and technology in the context of oral health. In today’s digital age, young adults represent a demographic that extensively engages with technology in other areas of their health, such as pedometers to track steps [46], heart rate monitors and activity trackers [47]. This study supported this notion by revealing that students were actively using apps and technology to monitor steps, exercise and track calorie intake. Many students also expressed that their parents cared about their oral health, suggesting a possible link that could be explored further, as research showed that parents frequently motivated young individuals to use software and devices designed to promote physical fitness and health [44]. Additionally, considering the increased positive outcomes of health promotion initiatives through technology [47] and the prevalent use of technology among young adults [44], it is noteworthy to recognise technology’s potential in relation to oral health.

Moreover, students acknowledged the pivotal role played by manual dexterity and coordination in establishing an efficient oral hygiene routine. Previous studies linked reduced manual dexterity to increased dental biofilm accumulation on teeth, suggesting a connection between poor manual dexterity and poor oral hygiene [48]. Students recognised the importance of maintaining oral hygiene and understood its heightened importance influenced by interactions with peers. This finding correlated with research indicating that young adults valued connecting with peers for healthcare guidance and well-being support [44]. This aligned with existing literature highlighting the importance of friendships during the university transition, which aids in adapting to a new social setting [2]. Furthermore, peer support and encouragement, particularly concerning dental appearance, considerably contributed to adopting improved oral hygiene practices. Dental aesthetics and appearance were noted to be of substantial importance in social interactions [49], impacting self-esteem and willingness to smile and communicate [50]. There exists an association between positive self-perception of oral health and satisfaction with one’s appearance [51].

Data analysis revealed crucial factors for optimal oral health behaviours in university students: (a) comprehensive knowledge, (b) essential skills like flossing, (c) timely reminders, (d) flexibility in routines, (e) awareness of peer challenges, (f) sensitivity to social perceptions, (g) recognition of negative outcomes, (h) positive behaviour perception with room for improvement, (i) enduring awareness of oral hygiene importance, (j) varied brushing habits, (k) likelihood of omitting certain practices when busy, (l) preference for continuity with previous dentists, (m) financial constraints affecting dental care, (n) mixed outcomes of accommodation on routines, (o) acknowledgement of workload impact, (p) changes in routines influenced by university life, including concerns about junk food and budget constraints.

### Strengths

In future interventions aimed at enhancing and improving oral health behaviours among students, this study provided insight into both the levels for applying behaviour change strategies and the operating components (BCTs) available for intervention designers to select and implement practically at appropriate levels. Additionally, creating, applying, and analysing all interview data using a methodical and organised framework such as the TDF and BCW allowed for a theory-driven examination of potential obstacles and supports in attaining optimal oral health behaviours. Finally, the utilisation of digital platforms, such as Zoom, revolutionised the way we communicated and enhanced accessibility. Also, communication with participants regarding scheduling ensured their ability to engage and participate at a time convenient to them.

### Limitations

The study’s sample size (*n* = 21) could be perceived as a limitation. However, recruitment continued alongside data collection, ending when data saturation was reached to ensure comprehensive results. As Green and Thorogood et al. (2004) noted, sample size should be based on when no further valuable data can be obtained. The use of an interview guide reflecting the TDF domains fostered a structured rather than fluid discussion atmosphere during interviews, potentially posing a limitation. However, this potential constraint was mitigated by establishing a strong rapport beforehand, thus creating a comfortable environment for participants to express themselves freely. Participants were also encouraged to discuss any pertinent aspects of their oral health behaviours outside the guide’s scope [52].

### Implications for future work

In terms of the COM-B model, the study identified many targets for a behaviour change intervention aimed at achieving optimal oral health behaviours in university students, including physical and psychological capability, physical and social opportunity, and reflective and automatic motivation. Moreover, specific TDF domains were identified as important, as indicated by participants’ interviews. Intervention functions such as education, enablement, training, environmental restructuring, and persuasion, were deemed appropriate for fostering optimal oral health behaviours. This study outlined 23 possible BCTs derived from this process. The APEASE (Acceptability, Practicability, Effectiveness, Affordability, Safety, and Equity) criteria should be used further to identify the most relevant intervention functions for this population. This criteria will help narrow down potential intervention functions [53]. By using this criteria, researchers can assess feasibility issues before implementing an intervention and look beyond the BCW.

## Conclusion

This study aimed to understand oral health behaviours and explore strategies to support university students in establishing and following optimal oral health behaviours. By achieving the study’s objectives – exploring barriers and facilitators using the TDF, identifying necessary changes through the BCW, and pinpointing specific BCTs for future interventions – it became clear that many university students lacked sufficient knowledge and awareness of oral hygiene practices during their transition to independence. Various influences, both positive and negative, created numerous barriers and facilitators to achieving optimal oral health behaviours. Informing students of oral health, providing dental products to alleviate financial restrictions, and helping and supporting them to plan schedules and prioritise would benefit students at university. The TDF and BCW offered a systematic approach to comprehending the determinants of behaviours in this demographic. By utilizing these frameworks, an intervention can be developed to achieve optimal oral health behaviours. When designing interventions for this population, it is essential for intervention developers to consider the factors within the BCW and BCTs to successfully promote and sustain improved oral health behaviours.

## Data Availability

The datasets used and/or analysed during this study are available from the corresponding author upon reasonable request.

## Abbreviations

BCW: Behaviour Change Wheel
BCTs: Behaviour Change Techniques
TDF: Theoretical Domains Framework
COM-B model: Capability, Opportunity, Motivation – Behaviour model
UREC: University Research Ethics Committee
PPI: Patient and Public Involvement
APEASE: Acceptability, Practicability, Effectiveness, Affordability, Safety, and Equity
